# A Mixed Methods Study Exploring Older Womens' Attitudes Toward Osteoporosis Medications: Adapting a Health Communication Framework

**DOI:** 10.1089/whr.2019.0014

**Published:** 2020-04-27

**Authors:** Lindsay N. Fuzzell, Liana Fraenkel, Susan L. Stark, Sarabjeet S. Seehra, Christine Nelson, Audrey Keleman, Mary C. Politi

**Affiliations:** ^1^ Department of Surgery, Washington University School of Medicine in St. Louis, Saint Louis, Missouri, USA.; ^2^ Department of Health Outcomes and Behavior, H. Lee Moffitt Cancer Center, Tampa, Florida, USA.; ^3^ Department of Rheumatology, Yale University School of Medicine, New Haven, Connecticut, USA.; ^4^ Department of Occupational Therapy, Washington University School of Medicine in St. Louis, Saint Louis, Missouri, USA.; ^5^ Applied Health Behavior, Washington University School of Medicine in St. Louis, Saint Louis, Missouri, USA.

**Keywords:** bisphosphonates, decision making, health communication, osteoporosis

## Abstract

***Background:*** Bisphosphonates (BPs) can reduce fracture risk for adults with osteoporosis (OP), but they have rare risks, complicating decision making. Guided by an established health decision and communication framework, we explored older women's feelings and positions toward taking BPs.

***Materials and Methods:*** Using a mixed-methods design, we interviewed women >65 years of age who had never taken BPs. After learning about BPs, participants responded to items about their feelings toward medication benefits/risks. They then identified their overall position toward taking BPs (corresponding to Unquestioning Acceptors, Cautious Acceptors, Hesitants, Probable Refusers, and Definite Refusers). We analyzed data using qualitative content analysis and summarized quantitative data with descriptive statistics.

***Results:*** Thirty women participated. Acceptors (*N* = 17, 56.6%) worried about OP-related fractures. Hesitant participants (*N* = 12, 40%) worried about BP risks, yet expressed openness toward medications if given opportunities to gather information and talk to clinicians. One Refuser expressed distrust in clinicians and pharmaceuticals.

***Conclusions:*** Understanding women's positions toward BPs might improve decision-making processes for OP treatment. Clinicians could tailor communication based on patients' identified BP position. Acceptors might be comfortable with succinct conversations describing medications. Hesitant patients might need more information from resources such as decision aids. Building trust with patients questioning BPs can support future conversations.

## Introduction

Osteoporosis (OP) is characterized by decreased bone mass and porous bones. Individuals with OP experience a higher risk of fracture than those with normal bone density.^1,2^ Over 12 million Americans will be diagnosed with OP by 2020,^3^ with women more frequently affected than men.^4,5^ OP-related hip fractures, vertebral fractures, and kyphosis (spine curvature) are associated with difficulty performing activities of daily living, reduced quality of life, chronic pain, and mortality.^6,7^ OP-related fractures in the United States may cost about 20 billion dollars annually.^8^

BPs are the most commonly prescribed medications for OP. BPs prevent further bone loss and decrease fracture risk.^9,10^ However, these medications can cause very rare, but serious, complications: atypical femur fracture,^11^ occurring in about 5 cases per 10,000 patient-years,^12^ and osteonecrosis of the jaw, occurring in <1 case per 100,000 patient-years.^13,14^ Patients also report side effects such as stomach and gastrointestinal discomfort.^15^ Despite the effectiveness of BPs and the rarity of severe side effects, use among adults with OP has declined by an estimated 20%–50%,^16,17^ due, in part, to patients' concerns about side effects.^18^ In a recent systematic review, after 1 year of BP use, persistence (time between initiation and discontinuation of medication) ranged from 28% to 74%.^19^

Decision making regarding BP use is complex. Adults may lack awareness of the seriousness of OP and the associated risk of fractures,^20–22^ and many are unaware of the morbidity and mortality risks associated with major fractures. Patients describe variability in their feelings about ease of decision making.^21^ Those with a positive trusting relationship with a clinician more often report a relatively easy decision-making process. Those turning to friends or family members for advice find the decision more complex, and may engage in more risk–benefit analysis while exploring concerns about BP side effects.^21^ Perceptions of medication benefit, distrust of pharmaceuticals, and concern about medication regimens can impact medication use over time.^23,24^ Among patients with recent fragility fractures who had begun taking BPs, some considered discontinuing medication as more time passed after fracture, or as BP side effects emerged, highlighting the fluidity of medication decision making.^22^

To improve primary adherence (filling a newly prescribed medication) and secondary adherence (refilling an existing prescription^25^) to BPs and decrease the burden of OP-related fractures, it is important to understand how women weigh the potential for rare, but serious, side effects of BPs against medication benefits. Other well-studied complex health decisions might enhance our understanding of BP decision making. Some medications that have substantial benefits, as well as risks, include anticoagulation medications for atrial fibrillation^26^ and thiazolidinediones to treat type 2 diabetes.^27^ These medications involve careful decision making so that patients and their health care teams can weigh substantial reductions in health risks with the introduction of potential side effects. In the literature examining vaccination decisions and related clinical communication, Leask et al. developed a framework categorizing parents' positions toward vaccinating their children according to their attitudes and readiness to accept vaccination.^28^ These categories included the Unquestioning Acceptor (parents “vaccinate…have no specific questions about safety and necessity”), Cautious Acceptor (“vaccinate…despite minor concerns”), Hesitant (“vaccinate but have significant concerns…are more focused towards risk”), Late/Selective Vaccinator (“delay or select only some vaccines”), and Refuser (“refuse vaccines…results from…existing philosophical position [or] negative experiences with the medical system…”).^28^ In this study, we examined whether similar positions may be applied to BP readiness.

This mixed methods study aimed to gain insight into how BP-naïve women without a known indication for BP therapy feel about them and their rare side effects, and how they weigh the risks and benefits of OP against potential medication side effects, to determine positions toward BPs. We adapted the vaccination position framework to explore how women's feelings about BPs impact their acceptance of medication.

## Materials and Methods

### Participants

Women aged 65 years and older who had never been offered and had never taken BPs were eligible to participate. Actual risk for fracture/bone mineral density *t*-score/indication for BP therapy was not criteria for eligibility and was not collected from participants. We chose to recruit treatment-naïve women because we expected they would be similar to women who are newly introduced to the medications during discussions with clinicians after a bone mineral density test. They would not have personal experiences with side effects or dosing requirements, allowing them to describe initial reactions to medication benefits and risks. Potentially eligible women were sent an e-mail through a list of volunteers interested in health research or were recruited through a local university clinic list of past research participants who agreed to be contacted for future studies. The first author (L.N.F.) screened potential participants *via* phone. Participants provided phone consent to complete eligibility and screening items and in-person consent to participate. We aimed to recruit ∼30 women for participation, continuing until we had reached concept saturation. The study was approved by the Human Research Protection Office of Washington University (#201801216).

### Procedures

We employed a mixed-methods approach to data collection, with participants completing both qualitative interviews and surveys. We developed a semistructured interview guide based on prior literature on OP and BP use,^29^ as well as the vaccination positions framework.^28^ Interviews focused on understanding women's knowledge and attitudes about OP, complications associated with it, and BP medications. Items focused on benefits of medications (*e.g.*, fracture risk reduction) and rare side effects (osteonecrosis of the jaw and atypical femur fracture). We asked participants to think about how they would feel about taking the medications if they were diagnosed with OP.

During each interview, information regarding OP and BPs was presented to participants using the latest standards in health risk communication and decision support to ensure all had an equivalent basis of knowledge. Accompanying particularly relevant items, participants viewed an image of kyphosis and related icon arrays (pictographs displaying number of expected vs. nonexpected events; iconarray.com). Icon arrays showed risk of outcomes for women 1 year after hip fracture, risk of further bone loss for women taking BPs, risk of fracture for women who do and do not take BPs. Participants also viewed a table showing benefits and possible harms associated with taking no medication compared with BPs. (See Appendices A1 and A2 for interview guide and benefits/harms table.) Interviews were conducted in a private room at the university or in participants' homes, depending on their preference. Two female members of the research team, trained in qualitative interviewing procedures (L.N.F. and C.N.), conducted interviews; each was completed in <1 hour.

After open-ended questions, women responded to items with ratings of their attitudes about OP medications. Twenty-one items on a 5-point Likert scale (strongly disagree to strongly agree) assessed feelings about OP and medications to treat it, including BPs. Based on the vaccine positions framework,^28^ participants were asked to respond to one item at the end of the interview that summarized their overall position toward taking BPs (see Table 1 for statements corresponding to each position category). They selected the statement that was most similar to their position. Participants also completed a short survey about their demographic and personal characteristics. They were given a $10 gift card for their time.

**Table 1.** Bisphosphonate Position Categories and Corresponding Statements Chosen by Participants

BP position category (categorized by researchers)	BP position statement (seen and self-selected by participants)
Unquestioning Acceptor	“If I had osteoporosis and my doctor recommended I take this medication, I would be willing to take it with no hesitation.”
Cautious Acceptor	“If I had osteoporosis and my doctor recommended that I take this medication, I would probably be willing to take it, but I am a little worried about it.”
Hesitant	“If I had osteoporosis and my doctor recommended that I take this medication, I'm not sure whether I would be willing to take it or not. I would have to think about it for a while. In some ways, I want to. In other ways, I don't.”
Probable Refuser	“If I had osteoporosis and my doctor recommended that I take this medication, I don't think I would be willing to take it.”
Definite Refuser	“If I had osteoporosis and my doctor recommended that I take this medication, I am certain that I would not be willing to take it.”

Position categories adapted from Leask et al.^28^

BP, bisphosphonate.

### Data analysis

Interviews were audio recorded, deidentified, and professionally transcribed. They were coded using QSR NVivo 11 and analyzed using content analysis. First, research team members (M.P. and L.N.F.) reviewed transcripts and developed a preliminary codebook based on the interview guide and emergent concepts within each of the broad BP position categories. Next, two team members (A.K. and S.S.S.) identified transcript segments addressing specific study questions and applied codes to those segments, beginning with the first five transcripts. Inconsistent codes were discussed and reviewed with the principal investigator (M.P.) to reach consensus. Once coders achieved inter-rater reliability (kappa ≥0.80, percent agreement ≥0.95), they independently coded a portion of the remaining 25 transcripts. Concepts describing BP positions were identified based on frequency and strength of statements within each position category. Qualitative findings are organized around position categories. Quotations are presented that exemplify each position category and highlight participants' perceived benefits and concerns about BP use.

Quantitative data were summarized with descriptive statistics and cross-tabulations using IBM SPSS Statistics 23. Responses to the 21 medication attitude items were trichotomized (disagree, uncertain, and agree).

## Results

### Participant characteristics

Sixty-five women were screened for eligibility. Thirty-three met eligibility criteria and were invited to participate, 32/33 (97.0%) consented/enrolled, 2 participants cancelled their interviews due to scheduling conflicts, and 30/32 (90.9%) completed in-person interviews. Participants were 73 years of age on average (standard deviation = 4.8, range = 66 − 86). Most were white, non-Hispanic (76.7%), and college educated (63.3%). About 47.0% reported an annual income <$40,000 (Table 2).

**Table 2.** Participant Characteristics (*N* = 30)

Participant characteristic	N (%) or mean (SD, range)
Age	72.7 (4.8, 66–86)
Race (*N*/% black or African American)	7 (23.3)
Education	
High school degree	5 (16.7)
Technical training or some college	6 (20.0)
College degree or higher	19 (63.3)
Household income	
Prefer not to answer	6 (20.0)
Less than $20,000	9 (30.0)
$20,000–$39,999	5 (16.7)
$40,000–$59,999	6 (20.0)
$60,000–$79,999	2 (6.7)
$80,000–$99,999	1 (3.3)
$100,000–$149,999	1 (3.3)

### BP positions

Table 3 summarizes participants' overarching position toward taking BPs should they be offered the medication, adapted from the Leask positions framework.^28^ On completion of interviews, 56.6% (*N* = 17) of participants endorsed a statement categorizing them as Acceptors (Unquestioning Acceptors 33.3%, *N* = 10; Cautious Acceptors 23.3%, *N* = 7), and 40.0% (*N* = 12) as Hesitant. Only one (3.3%) endorsed a statement characterizing herself as a Definite Refuser.

**Table 3.** Osteoporosis Medication Attitudes Responses by Bisphosphonate Position Category (*N* = 30)

	Unquestioning Acceptor (N = 10)			Cautious Acceptor (N = 7)			Hesitant (N = 12)			Definite Refuser (N = 1)		
	Agree, n (%)	Uncertain, n (%)	Disagree, n (%)	Agree, n (%)	Uncertain, n (%)	Disagree, n (%)	Agree, n (%)	Uncertain, n (%)	Disagree, n (%)	Agree, n (%)	Uncertain, n (%)	Disagree, n (%)
A. It makes sense to take BPs because the chances of breaking a bone from OP are much bigger than the chances of side effects from the medication.	9 (90)	0 (0)	1 (10)	5 (71)	0 (0)	2 (29)	8 (67)	3 (25)	1 (8)	0 (0)	0 (0)	1 (100)
B. I am just not interested in taking any medications for OP.	0 (0)	1 (10)	9 (90)	2 (29)	1 (14)	4 (57)	3 (25)	3 (25)	6 (50)	1 (100)	0 (0)	0 (0)
C. I would definitely take a medication to decrease the chances that my back could get hunched over.	9 (90)	0 (0)	1 (10)	6 (86)	1 (14)	0 (0)	8 (67)	1 (8)	3 (25)	0 (0)	0 (0)	1 (100)
D. I would definitely take a medication to decrease the chance that I could break a hip.	10 (100)	0 (0)	0 (0)	7 (100)	0 (0)	0 (0)	7 (58)	2 (17)	3 (25)	0 (0)	0 (0)	1 (100)
E. I just don't care enough about the chance of a broken bone to take a medication.	0 (0)	1 (10)	9 (90)	0 (0)	1 (14)	6 (86)	1 (8)	1 (8)	10 (83)	1 (100)	0 (0)	0 (0)
F. I am not willing to take a medication for OP.	0 (0)	0 (0)	10 (100)	1 (14)	0 (0)	6 (86)	2 (17)	2 (17)	8 (67)	1 (100)	0 (0)	0 (0)
G. I am not willing to accept any chance of side effects to treat OP.	1 (10)	0 (0)	9 (90)	1 (14)	0 (0)	6 (86)	1 (8)	1 (8)	10 (83)	0 (0)	1 (100)	0 (0)
H. I am willing to think about taking a medication to prevent my bone loss from getting worse.	10 (10)	0 (0)	0 (0)	7 (100)	0 (0)	0 (0)	11 (92)	0 (0)	1 (8)	0 (0)	0 (0)	1 (100)
I. I'm more scared of the side effects of BPs than of breaking a bone.	2 (20)	2 (20)	6 (60)	1 (14)	1 (14)	5 (71)	5 (46)	3 (25)	3 (25)	0 (0)	1 (100)	0 (0)
J. I am just too scared of getting either one of these side effects (jaw necrosis or atypical femur fracture) to take BPs.	1 (10)	0 (0)	9 (90)	1 (14)	0 (0)	6 (86)	2 (17)	2 (17)	8 (67)	0 (0)	1 (100)	0 (0)
K. If my doctor recommended that I take BPs, I would.	10 (100)	0 (0)	0 (0)	6 (86)	1 (14)	0 (0)	3 (25)	8 (67)	1 (8)	0 (0)	0 (0)	1 (100)
L. I have some doubts about whether it is necessary to take this medication (BPs).	4 (40)	0 (0)	6 (60)	5 (71)	0 (0)	2 (29)	5 (46)	4 (33)	3 (25)	1 (100)	0 (0)	0 (0)
M. I have some doubts about whether it's necessary to take any medication for OP.	0 (0)	1 (10)	9 (90)	0 (0)	2 (29)	5 (71)	4 (33)	3 (25)	5 (46)	1 (100)	0 (0)	0 (0)
N. I might initially agree with my doctor, but I'm not sure I would actually fill this prescription for BPs.	1 (10)	0 (0)	9 (90)	0 (0)	1 (14)	6 (86%)	6 (50%)	2 (17%)	4 (33%)	0 (0%)	0 (0%)	1 (100%)
O. BPs are known to have bad side effects. I'm not interested in taking them, but I would consider a new medication that was safer.	5 (50)	1 (10)	4 (40)	4 (57)	1 (14)	2 (29)	10 (83)	0 (0)	2 (17)	0 (0)	0 (0)	1 (100)
P. I don't understand why I should take a medication (like BPs) with side effects that only lowers your chance of a bone break by 50%.	2 (20)	1 (10)	7 (70)	2 (29)	1 (14)	4 (57)	6 (50)	2 (17)	4 (33)	1 (100)	0 (0)	0 (0)
Q. I'm willing to take vitamins and exercise to prevent a bone break, but I really don't want to take any prescription medications.	3 (30)	1 (10)	6 (60)	4 (57)	0 (0)	3 (43)	8 (67)	3 (25)	1 (8)	1 (100)	0 (0)	0 (0)
R. I am worried about the cost of BPs.	4 (40)	1 (10)	5 (50)	0 (0)	1 (14)	6 (86)	3 (25)	1 (8)	8 (67)	0 (0)	0 (0)	1 (100)
S. Adding another medication to my routine is too difficult.	1 (10)	0 (0)	9 (90)	1 (14)	0 (0)	6 (86)	2 (17)	3 (25)	7 (58)	0 (0)	0 (0)	1 (100)
T. It makes no sense for me to take a medication for OP because I feel fine.^a^	0 (0)	0 (0)	8 (100)	2 (33)	0 (0)	4 (67)	1 (11)	2 (22)	6 (67)	0 (0)	0 (0)	0 (0)
U. I would rather worry about the chance of having a fracture than the side effects of BPs. ^a^	1 (13)	1 (13)	6 (75)	0 (0)	0 (0)	6 (100)	2 (22)	4 (44)	3 33)	0 (0)	0 (0)	0 (0)

^a^ For items T & U, total *N* = 23.

### Qualitative content analysis

#### Acceptors

Acceptors of BPs often made statements about their worry about OP complications. One participant expressed:

Thinning of bones… [osteoporosis is] a disease I don't want…Life changing…you can't do things you'd like to do. You're always afraid of falling. You have to be careful with everything. (#019, Cautious Acceptor)

A few women worried about loss of independence and ability to conduct activities of daily living after osteoporosis-related fractures:

[Women with a broken hip] may [go] into assisted living because of their feeble condition. They're no longer bathing like they should. They're no longer cleaning the house. They're no longer cooking and eating properly… (#017, Cautious Acceptor)

With having a broken hip, it limits independence. You may end up in a nursing home, that it may not heal right, or that I could die from it, be one of those few that actually would die from it… I don't want to take that chance. (#016, Unquestioning Acceptor)

Worries about OP and its potential consequences informed participants' acceptance of a medication to prevent bone density loss and fractures:

Who wants to have broken bones? If you can take a medication that stops it, I'm all for it…If anything can help me with any kind of osteoporosis… yeah, I'll take a drug to help it. (#016, Unquestioning Acceptor)

A few women referenced the importance of their physician's recommendation about medication:

If my doctor prescribed it [medication for osteoporosis] to me, told me it could help me, I'm going to take it. (#020, Unquestioning Acceptor)

However, even Acceptors mentioned concerns about potential BP side effects. BP concerns did not outweigh perceived benefits for this group, but were present:

The side effects cause me concern. However, the overall benefits are, I think, still better to just go ahead and take the medication and risk the side effects. (#014, Cautious Acceptor)

…any time you read these side effects it makes you [think], ‘well somebody is the 1 in 10,000’. I mean that's what you think about. And yet statistically, it's probably a good chance to take based on what could happen to you from breaking a bone [from osteoporosis]. (#024, Unquestioning Acceptor)

Overall, Acceptors appeared to be more concerned about OP complications than potential side effects associated with BPs.

#### Hesitant

Hesitant participants expressed conflicting concerns about OP-related fractures and potential for the rare but serious side effects of BPs. They placed more weight on the risk of side effects than the Acceptors. They also emphasized a need to gather more information to make an informed decision about medications.

As one participant expressed:

[The side effect of the jaw bone]…Well, that's a big negative. It's a side effect that is unusual…I'm not saying it would keep many people from doing it [taking bisphosphonates], because the odds are low. [Yet] we do become more sensitive of our mouth functioning and our teeth when we get older. (#030, Hesitant)

Another participant described her fear of atypical femur fracture, but felt the risk may be acceptable assuming it could be repaired:

I think it would be devastating if it happened to me, but I think that's something I could live with, if there was a way to put it back together… (#012, Hesitant).

One woman acknowledged her conflicting feelings about osteonecrosis of the jaw:

The jaw [necrosis]…it just sounds so painful… [though] the chances are pretty slim… I think if I was in the situation [with osteoporosis], I might look at it totally different… I might say, ‘I'll try anything’ (#007, Hesitant).

Some Hesitant participants expressed interest in choosing vitamins, supplements, exercise, or diet to prevent OP concerns before they began prescription medication:

I think there are some things between not taking any medicine and taking a prescription for osteoporosis, like some calcium supplements or some Vitamin D supplements, or dietary changes… (#015, Hesitant)

I would probably try to compensate rather than jumping immediately to a medication…At this time I'm relying on supplements and exercise and eating well… (#025, Hesitant)

A few Hesitant participants expressed potential willingness to take BPs should they be offered one in the future, with the caveat that they would need to gather more information to mitigate worries about medication side effects:

I would try to get as many questions answered as I could [from my doctor]…I would not go right over to the pharmacy and have it filled and start taking it. I would ask [my doctor] a lot of questions, and I would do some reading, and I would talk to the pharmacist. (#015, Hesitant)

I think knowledge is power and I think you do your own research and you talk to other people who may be on that medication…and utilize the medical doctor that I've been with for a length of time…[and]…a pharmacist can be invaluable… (#025, Hesitant)

I would investigate the drugs because I'd want to know the side effects and whether it was worth it. You don't want osteoporosis side effects, but then you don't want to be cracking your teeth either…I wouldn't do it without looking into it…. (#027, Hesitant)

Some Hesitant participants also referenced considering their personal risk for fracture and how it might change over time before deciding:

It depends on the severity and progress of my osteoporosis. If I had a good bone density test two years ago, and today I have one that shows some deterioration…I would suggest that we do another test in a year. (#030, Hesitant)

If the tests were showing what [bone density] level you were at, and [my doctor] felt that it was good to start something…if you were already pretty far down the line [I might take it..]…how bad it is…would probably make a difference to me as to how willing I would be to do medication. (#023, Hesitant)

Overall, those who identified as Hesitant expressed competing concerns about the consequences of OP and BP side effects. Although these participants understood the potential for side effects was unlikely, they were undecided in their position toward taking BPs. Some expressed a need to gather more information to make a decision, some conveyed they would defer treatment until their condition worsened, and others wished to try nonpharmacologic options first.

#### Definite Refuser

One participant identified as a Definite Refuser and conveyed an overall distrust in clinicians and for pharmaceuticals. She was generally concerned about taking all medications, was not willing to accept any risk of side effects, and was pessimistic about the efficacy of BPs, in particular. She said:

I have a strong bias against taking medication in general. I know that's part of it, but what I know about these medications is that it seems like there's a lot of side effects and problems and they're not very effective. That there's a 50% efficacy rate or something, which may or may not be true, but my understanding is that it's not like it's definitely going to help. It might help for statistical groups or whatever [but]… it's oversold on the benefit” (#006, Definite Refuser).

She also spoke about the likelihood of rare side effects, indicating there was no risk that she was willing to take:

I've had many experiences in which I was in that five percent [of people with a rare consequence]—I've had more experiences than I care to think about. I've learned to not discount [those experiences]—statistics don't mean anything if you're the one that gets it” (#006, Definite Refuser).

She alluded to a distrust of clinicians and prescribing behaviors, saying:

I frankly have come to believe that there's a checklist and they [doctors] get paid based on certain things that they do. I think writing prescriptions is on there, that they get to code that they did that” (#006, Definite Refuser).

As a result of distrust for clinicians and pharmaceuticals, she relayed the preventative steps she had already taken to prevent OP and avoid prescription medications in the future:

I know what direction my bone density is going. Even so, I feel like I do a lot to keep my muscles strong and I think that's protective even if the bones themselves are getting more and more brittle. I think the fact that I had strength and speed and agility and that sort of thing is protective (#006, Definite Refuser).

### OP medication attitudes

Table 3 displays participants' responses to 21 statements about OP medication attitudes. Overall, most Acceptors were more worried about complications of OP than the BP-specific side effects. Hesitant participants often answered they were “uncertain” and were less consistent in responses to attitude items. The single Refuser responded consistently across items, indicating disinterest in all pharmaceuticals and in any medication that could treat OP. Many participants' responses indicated they were worried about OP overall and were willing to take medication to treat it, but were unwilling to take BPs in particular because of concerns about side effects. For instance, most participants (24/30, 80%) disagreed with an item stating, “I am not willing to take a medication for osteoporosis.” Similarly, more participants (15/30, 50%) agreed with an item stating, “I have some doubts about whether it's necessary to take this medication (bisphosphonates)” compared with those (5/30, 17%) agreeing with the item “I have some doubts about whether it's necessary to take any medication for osteoporosis.” These responses suggest trepidation toward BPs rather than OP treatment in general. Hesitant participants often (5/12, 42%) indicated they were “more scared of the side effects of bisphosphonates than of breaking a bone from osteoporosis,” compared with Acceptors (2/10, 20%).

## Discussion

We adapted a vaccination position and communication framework^28^ for women who might be offered BPs to enrich our understanding of OP medication attitudes and willingness to take the medications. We used mixed methods to better understand participants' positions toward BPs. Overall, our findings may provide a novel way for clinicians to approach BP communication in the context of feelings toward the medications.

BP usage may not be right for all patients, despite effectiveness. Some might not be willing to accept tradeoffs between potential benefits and risks. A few short screening questions might help determine how patients initially feel about BPs; clinicians could guide patient-centered discussions based on patients' attitudes toward taking medication.^28^ For example, across all BP attitudes and positions, clinicians could build trust with patients, efficiently give information, and use a style consistent with motivational interviewing (*e.g.*, ask questions revealing medication readiness and motivation for fracture prevention).^28^ Some older adults might be flexible in their positions toward BPs based on change in bone mineral density or fractures.^22^ By building rapport and maintaining relationships with patients, clinicians support the possibility of future prevention and treatment acceptance for those flexible in their BP position.^28^

For Acceptors (the majority of our sample), it might be most helpful to provide verbal and numerical assessments of personalized OP risks and benefits to reinforce BP positions. Discussions can be succinct, while thoroughly answering questions about BPs as they arise. These strategies could also benefit Hesitant participants. However, because Hesitants expressed a need for more information, decision aids or educational brochures about medication tradeoffs may be used to supplement clinical conversations and help them weigh personal risks and benefits of BPs. For Refusers, the primary goal of communication could be to build trust and encourage future discussion about fracture reduction solutions through follow-up appointments, rather than discussing OP and BP risks upfront. Refusers might not be ready to have in-depth discussions about medication use, and clinicians must meet patients where they are in terms of prevention and treatment.

This study has several unique strengths. We used a mixed-methods design to assess participants' BP attitudes, which enabled us to identify endorsed BP positions and then further explore beliefs related to stated positions through both qualitative interview responses and closed-ended questionnaire items. We recruited women who were treatment naïve and had never taken BPs to avoid biases of those who were currently taking or had refused to take the medication in the past. We also applied an established framework to positions regarding BPs, a novel approach for assessing older adults' attitudes toward this treatment option. Participants' responses about their hypothetical BP acceptance corresponded with the existing framework, suggesting similarities in decision-making processes across medical contexts. Future research could test specific messages tailored for each position in larger quantitative studies, which would be well informed by prior work on primary and secondary adherence.^23,30,31^ Because this decision framework could be applicable to other complex medication decisions, research on the distribution of positions with these and other patient populations would be an interesting area for future investigation.

Findings should be interpreted in the context of study limitations. Although BP decisions can be viewed similarly to vaccination decisions because of the documented benefits and low likelihood of side effects,^32^ some differences are apparent. For example, there is a large amount of misinformation about vaccinations online^33–35^ and broad vaccine coverage in the media; these media biases might not be salient among older adults considering BPs.^36^ Vaccine refusers also put others at risk when they do not vaccinate their children,^32^ unlike older adults who defer BP use. In addition, the percentages of accepting, hesitant, and refusing participants in this study were not meant to reflect the true proportion of patients who accept or refuse BPs in practice^16^; we encountered only one participant who identified as a Refuser, limiting generalizability and capability to explore Refusers' attitudes compared with other participants' positions. Our Refuser's beliefs were consistent with prior research suggesting patients who never start, or stop BPs prematurely, may perceive fewer medication benefits, believe they have lower susceptibility to OP-related fractures, and have lower self-efficacy for medication use.^23^ Our refusing participant was also similar to other BP refusers who distrust medications and believe those in the medical community are over-reliant on prescribing them.^23^ Furthermore, we classified women's positions based on their response to a single item endorsed at the conclusion of their interview.

In addition, our sample was largely college educated and white. Participants in this study were also presented with balanced educational information about BPs (benefits/harms, images of kyphosis, and icon arrays) before responding to standardize information among a treatment-naïve group. However, this process might not reflect clinical practice patterns. It is important to note that participants were not making an actual medication decision and did not have a known fracture risk or indication for BP therapy. Receiving an OP diagnosis or being offered a BP by a clinician could lead to different feelings toward the medications. Future research is needed to better understand women's positions toward BPs, particularly among Refusers who represent a large proportion of women in practice. Research with treatment-naïve women providing feedback about perceptions of benefits and side effects, as well as with women diagnosed with OP and offered medications, would add to these findings.

## Conclusions

The position framework described in this study improves our understanding of how treatment-naive women approach the decision of whether to initiate BPs for OP. Positions identified could inform patient–clinician approaches to communication and education about BPs. For patients who appear to be initially accepting of BPs based on brief clinical interviewing, clinicians could provide brief descriptions of risks and benefits, and answer questions as needed. Hesitant patients might need more discussion and information about BPs to make a decision. For those who initially refuse BPs, clinicians should continue to build rapport and trust to encourage future discussion about fracture reduction solutions during follow-up appointments. Refusers may fear specific side effects, and clinicians must meet patients where they are with the goal of fracture prevention and/or future treatment through long-term care. Future research could test specific messages tailored to each of the positions in larger quantitative studies, and/or explore the impact of tailored messages on decision making among women already diagnosed with OP.

## Ethics Approval and Consent to Participate

The study was approved by the Human Research Protection Office of Washington University (#201801216). All participants provided verbal telephone consent and written in-person consent before participating in this study.

## Consent for Publication

All participants provided consent to publication within their written in-person consent.

## Availability of Data and Materials

Qualitative interview and quantitative data from this study can be made available from the senior author upon reasonable request.

## Disclaimer

The content of this article is solely the responsibility of the authors and does not necessarily represent the official views of the organizations supporting this study.

## Authors' Contributions

L.N.F. conducted qualitative interviews, developed a preliminary qualitative codebook, conducted quantitative analyses, wrote the first draft of the article, and edited subsequent drafts of the article. L.F. conceived of the study design, revised and approved of the qualitative codebook, and edited subsequent drafts of the article. S.S. conceived of the study design, revised and approved of the qualitative codebook, and edited subsequent drafts of the article. S.S.S. and A.N. coded qualitative interviews and reviewed drafts of the article. C.N. conducted qualitative interviews and reviewed drafts of the article. M.C.P. served as senior author, conceived of the study design, developed a preliminary qualitative codebook and revised/approved the final codebook, supervised qualitative coding, and edited subsequent drafts of the article. All authors read and approved the final article.

## Abbreviations Used

BPs: bisphosphonates
OP: osteoporosis

## Appendix

### Appendix A1. Interview Guide

**Thank you for participating in this study. We are hoping to learn what you think about a condition called osteoporosis and options to treat it. We would like to get a sense of what you think so that we can help other women with osteoporosis learn about ways to prevent broken bones (or fractures). We know thinking about this can be overwhelming. We are doing the study to understand how women in general feel about osteoporosis and its medications. If you have questions about your own bone health, you can ask your health care provider.**

**OSTEOPOROSIS**

1. **Have you heard of osteoporosis?**

If yes: **What have you heard about osteoporosis?**

If no/unsure/to clarify: **Osteoporosis is a condition in which the bones become weak. People with osteoporosis have a higher chance of breaking bones. Broken bones can happen anywhere but are most common in the back, wrist, and hip.**

2. **What comes into your mind when you think about osteoporosis?**

If participant does not discuss consequences: **More specifically, what comes into your mind when you think about what can happen if you have osteoporosis?**

If participant does not discuss consequences**: *Broken bones can happen anywhere, but are most common in the back, wrist, and hip.***

**What do you picture (in your mind) when you think about having a broken wrist?**

**What do you picture (in your mind) when you think about having a broken hip?**

If need prompt: **What do you think life would be like after breaking a bone in your hip?**

**When you look to see where people are 1 year after they have fallen and broken their hip: 65% were able to go back to living in the community**

**20% had to stay in a nursing home**

**15% had died**

**[Show icon array.] What comes to mind when you hearing these things about breaking a bone in your hip?**

**Optional** if patient mentions death statistic: We don't really know why breaking a hip bone sometimes leads to death. It might be that some people lose the ability to live on their own. It could be that they have less ability to walk around and do things that keep them healthy. It also could be that older people who break a bone are also likely to be more frail.

**If you have several broken bones in your back because of osteoporosis**, **you may get kyphosis. Kyphosis is forward rounding of the back that creates a hunchback appearance. Here is a picture of what kyphosis looks like. (Show picture of kyphosis.)**

**Overall, what comes into your mind when you think about the chance of getting a curved back, like the one in the picture?**

*************************************************************************************************************

3. **Have you heard about medications (bisphosphonates such as Fosamax, Boniva, or Reclast) used to treat osteoporosis?**

If yes: **Where or from whom have you heard of them (*e.g.*, doctor, TV or radio commercial, word of mouth)?**

Optional: **What has your doctor told you about these medications? Did she or he recommend that you start taking the meds?**

If no/unsure/to clarify: **Medications are available that prevent further bone loss and lower the chance of breaking a bone.**

**If your doctor recommended that you took a medication to treat osteoporosis to lower the chance of these bone breaks, how would you feel?**

**************************************************************************************************************

**BENEFITS AND RISKS**

**Now we are going to talk about some benefits and possible harms of these osteoporosis medications. Keep in mind that women usually take them for about 3–5 years.**

**I'm going to show you a table that displays the benefits and possible harms of these osteoporosis medications. Please take a minute to look this over. [Read with the participant to orient them to the table.]**

4. **A.What is the first thing (either a benefit or harm) that stands out to you in this table?**
  **i. Prompts if needed: What grabs your attention about this? What is your initial reaction to learning this particular fact? Would this make you more or less likely to take the medication if you had osteoporosis?**
  **B. What is your initial reaction to learning about the benefits of these medications compared with the possible harms? Do you think the benefits outweigh the possible harms? Why or why not?**
  **C. What is your gut reaction to learning about this rare side effect of an exposed jaw bone? [If they ask questions about osteonecrosis of the jaw: Most patients who get this problem can be treated without surgery. Taking care of your mouth and going to the dentist every year is the best way to prevent this from happening.]**
  **D. What is your gut reaction to hearing about a chance of an unusual break of the thigh bone? [If they ask questions about atypical femur facture: These unusual breaks are different than the type of break that happens from osteoporosis without medication, and are usually called “atypical femur fractures.”]**

**Optional** if participant asks *why* the medicine causes femoral fractures: It is very frustrating that a medication that decreases the chance of breaking a bone may also be linked to a break in the thigh bone. We do not know why people can break a thigh bone while on the medicine. We just know there is a small link between the medicine and breaking this type of bone. It is important to remember that the medication prevents many more fractures than it may cause.

5. **Overall, how would you feel about taking these types of osteoporosis medications after seeing both the benefits and harms of the medications?**

If need prompt: **Overall, how do you feel about the chances of breaking a bone compared to the chances of side effects from the medications?**

**Optional** if participant asks about other minor side effects: Some people talk about stomach problems, but research found that people taking the medicine do not have stomach problems more than people taking a placebo (or sugar pill).

6. **Osteoporosis Medication Attitudes**

For these items: **Imagine you were diagnosed with osteoporosis.**

For those with and without osteoporosis, read one statement at a time. Rate on scale (1 [strongly disagree] to 5 [strongly agree]) and follow each item with a probe as to why they chose the number they chose (*e.g.*, “Can you tell me WHY you feel this way?”).

A. It makes sense to take the medication (bisphosphonates) because the chances of breaking a bone from osteoporosis are much bigger than the chances of the side effects from the medication.
B. I am just not interested in taking any medications for osteoporosis.
C. I would definitely take a medication to decrease the chances that my back could get hunched over.
D. I would definitely take a medication to decrease the chance that I could break a hip.
E. I just don't care enough about the chance of a broken bone to take a medication.
F. I am not willing to take a medication for osteoporosis.
G. I am not willing to accept any chance of any side effects to treat osteoporosis.
H. I am willing to think about taking a medication to prevent my bone loss from getting worse.
I. I'm more scared of the side effects of bisphosphonates than of breaking a bone.
J. I am just too scared of getting either one of these side effects (jaw necrosis or atypical femur fracture) to take the medication (bisphosphonates).
K. If my doctor recommended that I take this medication (bisphosphonates), I would.
L. I have some doubts about whether it is necessary to take this medication (bisphosphonates).
M. I have some doubts about whether it's necessary to take **ANY** medication for OP.
N. I might initially agree with my doctor, but I'm not sure I would actually fill this prescription for osteoporosis.
O. These medications (bisphosphonates) are known to have bad side effects. I'm not interested in taking them, but I would consider a new medication that was safer.
P. I don't understand why I should take a medication (like bisphosphonates) with side effects that only lowers your chance of a bone break by 50%.
Q. I'm willing to take vitamins and exercise to prevent a bone break, but I really don't want to take any prescription medications.
R. I am worried about the cost of the medication (bisphosphonates).
S. Adding another medication to my routine is too difficult.
T. It makes no sense for me to take a medication for osteoporosis because I feel fine.
U. I would rather worry about the chance of having a fracture than the side effects of this medication (bisphosphonates).

7. **Are there other reactions/thoughts/feelings you have about osteoporosis or its treatment, or that you think other women might have?**

************************************************************************************************************

8. **How well would a medication for osteoporosis have to work for you to be ok with taking it?**

**Currently, medications decrease the chances of breaking a bone by about half. How much better would this have to be for you to be willing to take a medication?**

9. **Is there any risk of a side effect of the jaw that you would accept?**
10. **Is there any risk of unusual break of the thigh bone that you would accept?**
11. **After thinking about all of this, do you agree with any of these descriptions:**

(Show the participant each statement, but not the label that goes with it.)

Ask the participant to choose which statement best fits with how they feel.

A. ***Unquestioning Acceptor*: If I had osteoporosis and my doctor recommended that I take this medication, I would be willing to take it with no hesitation.**
B. ***Cautious Acceptor*: If I had osteoporosis and my doctor recommended that I take this medication, I would probably be willing to take it, but I am a little worried about it.**
C. ***Hesitant*: If I had osteoporosis and my doctor recommended that I take this medication, I'm not sure whether I would be willing to take it or not. I would have to think about it for a while. In some ways, I want to, in other ways, I don't.**
D. ***Probable Refuser*: If I had osteoporosis and my doctor recommended that I take this medication, I don't’ think I would be willing to take it.**
E. ***Definite Refuser*: If I had osteoporosis and my doctor recommended that I take this medication, I am certain that I would not be willing to take it.**

**Or is there anything else you would like to add that better describes how you feel/would feel about taking medication for osteoporosis?**

### Appendix A2. Bisphosphonate Benefits and Harms Table

**Osteoporosis Medication (Bisphosphonates): Yes or No?**

Osteoporosis is a condition in which the bones become weak. People with osteoporosis have a higher chance of breaking bones. Broken bones can happen anywhere, but are most common in the back, wrist, and hip. All people with osteoporosis should exercise, get enough calcium and vitamin D, and be careful when walking or climbing stairs to lower the risk of falling. Bisphosphonate (BP) medications are also recommended because they lower the risk of breaking a bone, even in women who exercise, get enough calcium and vitamin D, and try their best to avoid falling. Use this decision tool to help you think about whether or not you would take BPs such as Fosamax, Boniva, or Reclast if you are diagnosed with osteoporosis.

**Table:**

Frequently asked questions ↓	BP medications	No medication
**Who is it recommended for?**	People with osteoporosis (weak bones)	People without osteoporosis. People who have osteoporosis plus kidney disease
**What are the benefits?**	**Lowers chance of breaking a bone**, 20 in 100 women with osteoporosis break a bone without taking medication, and only 10 in 100 who take this medication break a bone.	**Avoids possible side effects of medications.**
	**Lowers chance of forward curve of the spine (kyphosis), disability, and loss of independence.**	
**What are some harms that could happen?**	This medication has very rare side effects such as:	**Bone breaks in the back, hip, or wrist**. Breaks in back bones can lead to curve of spine. Breaks in a hip bone can lead to disability. About 35 in 100 will no longer be able to live on their own after breaking a hip.
	**A problem with the jaw bone, where the lower or upper jaw is exposed.** This happens in 1 in 10,000 to 1 in 100,000 people.	
	**An unusual break of the thigh bone.** This happens in about 1 in 10,000 people.	
